# Comparison of Adverse Event Profiles of Amphotericin B Formulations Using Real-World Data

**DOI:** 10.7759/cureus.71588

**Published:** 2024-10-16

**Authors:** Yuka Nokura, Mika Maezawa, Koumi Miyasaka, Sakiko Hirofuji, Satoshi Nakao, Moe Yamashita, Nanaka Ichihara, Kana Sugishita, Tomofumi Yamazaki, Hirofumi Tamaki, Kazuhiro Iguchi, Kohei Tahara, Mitsuhiro Nakamura

**Affiliations:** 1 Laboratory of Drug Informatics, Gifu Pharmaceutical University, Gifu, JPN; 2 Department of Pharmacy, Kyushu University Hospital, Fukuoka, JPN; 3 Laboratory of Community Pharmacy, Gifu Pharmaceutical University, Gifu, JPN; 4 Laboratory of Pharmaceutical Engineering, Gifu Pharmaceutical University, Gifu, JPN

**Keywords:** ambisome, amphotericin b, fda adverse event reporting system, fungizone, liposomal amphotericin b

## Abstract

Amphotericin B deoxycholate (AMPH-B) is a polyene macrolide with antifungal activity. Liposomal AMPH-B (L-AMB) was developed to reduce side effects while maintaining antifungal activity. This study was aimed at evaluating and comparing the adverse event profiles of AMPH-B and L-AMB using a spontaneous reporting system.

We analyzed the adverse event reports of AMPH-B and L-AMB from the United States Food and Drug Administration Adverse Event Reporting System (FAERS). Case report counts of adverse events were generated according to the preferred terms of the Medical Dictionary for Regulatory Activities (MedDRA). Standardized MedDRA queries (SMQs) and system organ classes (SOCs) were used to compare the organ-specific adverse event profiles of AMPH-B and L-AMB. The reporting odds ratio and proportional reporting rate were used to detect pharmacovigilance signals.

The FAERS database contains 21,173,818 cases from January 2004 to March 2024. Adverse events were reported in 2438 cases receiving AMPH-B treatment and 3344 cases receiving L-AMB treatment, including 848 and 1591 cases receiving intravenous AMPH-B and L-AMB injections, respectively. The most frequently reported drug-related adverse event in the AMPH-B and L-AMB groups was hypokalemia. SOCs with statistically significant differences were “Inv” (laboratory tests), “Resp” (respiratory, thoracic, and mediastinal disorders), “Genrl” (general and systemic disorders and conditions at the site of administration), “Card” (cardiac disorders), and “Blood” (blood and lymphatic system disorders). No statistically significant difference was observed in the SMQ profile of adverse events in “Renal” (renal and urinary disorders) and “Hepat” (hepatobiliary disorders) between the L-AMB and AMPH-B formulations in this study.

Based on real-world data from FAERS, adverse event profiles of AMPH-B and L-AMB were compared. No statistically significant difference was observed in the SMQ profile of adverse events in the renal and hepatic SOCs between the L-AMB and AMPH-B formulations. Our results suggest that L-AMB is more tolerated by the kidneys than AMPH-B.

## Introduction

Amphotericin B deoxycholate (AMPH-B) is a polyene macrolide with antifungal activity. It is used in an oral form to treat abnormal *Candida* growth in the gastrointestinal tract and in an injectable form to treat deep-seated dermatomycoses and fungal infections, such as *Aspergillus*, *Candida*, *Cryptococcus*, *Mucor*, and *Coccidioides* infections. For example, Fungizone® is an injectable AMPH-B formulation that has been used as the gold standard for the treatment of deep mycosis for more than 60 years since its launch in 1962. However, AMPH-B injections cause fever, chills, and many other serious side effects, including hypokalemia and renal dysfunction [1-3].

Recently, new drug delivery systems have been developed to increase the therapeutic efficacy of drugs and reduce their organ toxicity. Liposomalization is a technique used to develop such systems to either increase drug concentrations in tumor cells or decrease drug exposure in normal tissues. Liposomes are closed spherical vesicles consisting of multiple concentric bilayers of phospholipids, cholesterol, and other affinity materials mixed in specific proportions [4,5]. For example, liposomal amphotericin B (L-AMB) was developed to reduce serious side effects, while maintaining efficacy. The L-AMB Ambisome® showed the same pharmacological activity as AMPH-B in vitro against *Aspergillus fumigatus*, *Aspergillus flavus*, *Candida albicans*, *Candida krusei*, *Candida lusitaniae*, *Candida parapsilosis*, *Candida tropicalis*, *Cryptococcus neoformans*, and *Blastomyces dermatitidis* [4].

In an examination of the cytotoxicity of AMPH-B on human erythrocytes by measuring hemolytic potential, a difference of approximately 100-fold was observed between AMPH-B and L-AMB [6]. The cytotoxicity of L-AMB in some cell lines was also reported to be lower than that of AMPH-B [6]. Although different dosage forms of AMPH-B and L-AMB are expected to have different adverse event profiles, their descriptions in the package inserts are not quantitative [3,4], and few reports comprehensively evaluate the various adverse event profiles of different dosage forms.

L-AMB generally induces lesser adverse events than AMPH-B [4,5], with lowered risk of kidney damage [7]. However, the possible difference between the renal impairment profiles of L-AMB and AMPH-B has not been evaluated in diverse clinical settings. Although the advantage of L-AMB over AMPH-B is widely accepted, we believe that without this knowledge, there may be a risk of missing adverse events induced by L-AMB in routine practice. However, to our knowledge, few studies have compared the adverse event profiles of different AMPH-B formulations.

The United States Food and Drug Administration (FDA) Adverse Event Reporting System (FAERS) is the world’s largest spontaneous reporting system (SRS); it collects a wide variety of adverse drug events from clinicians and pharmaceutical companies and is recognized as a major pharmacovigilance tool that closely reflects clinical practice [8-10]. To our knowledge, few comprehensive studies have investigated the adverse events associated with AMPH-B and L-AMB using FAERS. Therefore, this study aimed to evaluate and compare the adverse event profiles of AMPH-B and L-AMB using data from the FAERS database.

## Materials and methods

Ethical approval was not sought for this study because it was an observational study without any participants. All the results were obtained from data openly available online on the Food and Drug Administration website. All data from the FAERS database were fully anonymized by the regulatory authorities before we accessed them.

Data from the FAERS database were downloaded from the FDA website [11]. The FAERS database structure conforms to the international safety reporting guidelines drafted by the International Council on Harmonization (E2B). The FAERS database comprises seven data tables: patient demographic and administrative information (DEMO), drug/biological information (DRUG), adverse events (REAC), patient outcomes (OUTC), report sources (RPSR), drug therapy start and end dates (THER), and indications for use or diagnosis (INDI).

We created our dataset from the FAERS database using FileMaker Pro 18 Advanced software (FileMaker, Inc., Santa Clara, CA, USA), according to the ASCII Entity Relationship Diagram, which is publicly available on the FDA website. According to FDA recommendations, we removed duplicate reports from the same patient in the FAERS database from the analysis. Drugs reported in the case reports were classified into four categories according to the degree to which they were expected to contribute to adverse events: primary suspect (PS), secondary suspect (SS), concomitant (C), and interacting (I) drugs. Drugs recorded as PS were used for the analysis. Drugs in the FAERS database are registered voluntarily. They can be registered using generic names, brand names, or abbreviations. DrugBank (The Metabolomics Innovation Centre, Canada) is a reliable drug database that is used as a reference for pharmacovigilance analysis [12]. In this study, it was used as a source for batch conversion and drug name integration.

Case report counts of adverse events were generated according to the preferred terms (PTs) of the Medical Dictionary for Regulatory Activities (MedDRA) version 27.0. Standardized MedDRA queries (SMQs) are widely used to analyze SRS reports. They were constructed by the Maintenance and Support Services Organization and group PTs according to the levels associated with defined medical conditions. SMQ and system organ classes (SOCs) were used to compare the organ-specific adverse event profiles of AMPH-B and L-AMB. Pearson’s chi-square test was used to compare data between the two formulations. Data were considered statistically significant at p<0.05.

We calculated the reporting ratio and the reporting odds ratio (ROR) to study the influence of AMPH-B (Fungizone®) and L-AMB (Ambisome®) on adverse events [8-10,13]. A 2×2 contingency table was then created to identify drug combinations resulting in disproportionately adverse events. The “cases” were defined as patients reporting adverse events after AMPH-B and L-AMB use, and “non-cases” were defined as patients reporting all other events. ROR values were calculated as (a × d)/(b × c) and expressed as point estimates with 95% confidence intervals (CIs). A signal was defined as the lower limit of the 95% CI of the ROR being greater than one. Two or more cases are required to identify a signal [8,14].

All data analyses were performed using JMP Pro 16 software (JMP Statistical Discovery, Cary, NC, USA).

## Results

The FAERS database contains 21,173,818 cases from January 2004 to March 2024. We analyzed 17,714,041 reports, excluding duplicate reports according to FDA recommendations. Adverse events of AMPH-B and L-AMB were reported in 2438 and 3344 cases, respectively; of these cases, 848 and 1591 cases received intravenous injections of AMPH-B and L-AMB, respectively. The most frequently reported adverse event related to AMPH-B was hypokalemia (excluding the PTs, “DRUG INEFFECTIVE” and “OFF LABEL USE”), followed by pyrexia and renal impairment. The most frequently reported adverse event related to L-AMB was hypokalemia (excluding the PTs, “DRUG INEFFECTIVE” and “OFF LABEL USE”), followed by renal impairment and dyspnea. No significant difference was observed in the expression profile of ROR signals between the two formulations of AMPH-B and L-AMB (Tables 1, 2).

**Table 1:** Number of reports and reporting odds ratios for amphotericin B formulations (AMPH-B) ^*^ Confidence Interval

Preferred term code	Preferred term	Total	Case (n)	Reporting odds ratio (95% CI^*^)
10013709	Drug ineffective	1128032	224	5.3 (4.5–6.1)
–	Off label use	650743	116	4.2 (3.4–5.1)
10021015	Hypokalemia	39093	47	26.6 (19.8–35.6)
–	Death	730753	38	1.1 (0.8–1.5)
10037660	Pyrexia	299290	38	2.7 (2.0–3.8)
10062237	Renal impairment	70466	30	9.2 (6.4–13.2)
–	Chills	102188	29	6.1 (4.2–8.8)
10043071	Tachycardia	76652	29	8.2 (5.6–11.8)
10069339	Acute kidney injury	125590	28	4.8 (3.3–7.0)
10005483	Blood creatinine increased	57873	25	9.3 (6.2–13.8)
–	Condition aggravated	240861	23	2.0 (1.3–3.1)
10013968	Dyspnea	486578	23	1.0 (0.7–1.5)
10077361	Multiple organ dysfunction syndrome	20960	20	20.4 (13.1–31.8)
10051792	Infusion-related reaction	52388	18	7.3 (4.6–11.7)
10033661	Pancytopenia	47122	18	8.1 (5.1–13.0)
10035664	Pneumonia	271408	17	1.3 (0.8–2.1)
10007515	Cardiac arrest	73279	17	4.9 (3.0–8.0)
10043089	Tachypnea	11352	17	31.9 (19.8–51.7)
10038435	Renal failure	122048	17	2.9 (1.8–4.8)
10033318	Oxygen saturation decreased	45423	16	7.5 (4.6–12.3)
–	Renal failure acute	43092	16	7.9 (4.8–12.9)
10038695	Respiratory failure	63850	16	5.3 (3.2–8.7)
10040070	Septic shock	36043	14	8.2 (4.9–14.0)
10020646	Hyperkalemia	29887	14	9.9 (5.9–16.9)
10021097	Hypotension	173419	14	1.7 (1.0–2.9)
10043554	Thrombocytopenia	94721	13	2.9 (1.7–5.0)
10051118	Drug ineffective for unapproved indication	43837	13	6.3 (3.6–10.9)
10029354	Neutropenia	112363	13	2.4 (1.4–4.2)
–	Disease progression	98477	13	2.8 (1.6–4.8)
10029155	Nephropathy toxic	8956	13	30.8 (17.8–53.3)
10037844	Rash	358880	12	0.7 (0.4–1.2)
10021027	Hypomagnesemia	11470	12	22.2 (12.5–39.2)
–	Headache	541085	12	0.5 (0.3–0.8)
10005724	Blood potassium decreased	26225	12	9.7 (5.5–17.1)
10008479	Chest pain	163743	12	1.5 (0.9–2.7)
10047700	Vomiting	396955	11	0.6 (0.3–1.0)
10020772	Hypertension	182177	11	1.3 (0.7–2.3)
10028813	Nausea	674394	11	0.3 (0.2–0.6)
10020751	Hypersensitivity	159049	11	1.5 (0.8–2.6)
10027417	Metabolic acidosis	26468	10	8.0 (4.3–14.9)
10044565	Tremor	146245	9	1.3 (0.7–2.5)
10038687	Respiratory distress	24185	9	7.8 (4.1–15.1)
10040047	Sepsis	96944	9	1.9 (1.0–3.8)
10017533	Fungal infection	28546	9	6.6 (3.4–12.8)
–	Drug interaction	138348	9	1.4 (0.7–2.6)
10027091	Medication error	47854	9	4.0 (2.1–7.6)
10006473	Bronchopulmonary aspergillosis	6385	9	29.8 (15.4–57.5)
10021143	Hypoxia	29665	9	6.4 (3.3–12.3)
10003988	Back pain	202606	8	0.8 (0.4–1.7)
10006482	Bronchospasm	12639	8	13.3 (6.7–26.8)
10014387	Electrocardiogram QT prolonged	30412	8	5.5 (2.8–11.1)
10074171	Aspergillus infection	5097	8	33.1 (16.5–66.5)
10020642	Hyperhidrosis	113791	8	1.5 (0.7–3.0)
10038537	Renal tubular disorder	2575	8	65.7 (32.7–132.0)
10038428	Renal disorder	40451	8	4.2 (2.1–8.3)
10003119	Arrhythmia	42283	8	4.0 (2.0–8.0)
10000880	Acute myeloid leukemia	13325	8	12.7 (6.3–25.4)
10065042	Immune reconstitution inflammatory syndrome	4357	8	38.8 (19.3–77.9)
10065553	Bone marrow failure	19108	8	8.8 (4.4–17.7)
10028098	Mucormycosis	2113	7	70.0 (33.2–147.5)
10019663	Hepatic failure	26599	7	5.5 (2.6–11.6)
10038540	Renal tubular necrosis	8395	7	17.6 (8.3–37.0)
10051082	Therapy non-responder	33407	7	4.4 (2.1–9.3)
–	General physical health deterioration	91284	7	1.6 (0.8–3.4)
–	Multi-organ failure	17736	7	8.3 (3.9–17.5)
10002198	Anaphylactic reaction	44463	7	3.3 (1.6–7.0)
10071408	Maternal exposure during pregnancy	68553	7	2.1 (1.0–4.5)
10029147	Nephrogenic diabetes insipidus	1008	7	147.3 (69.8–310.7)
10011224	Cough	234271	7	0.6 (0.3–1.3)
10005911	Body temperature increased	18329	7	8.0 (3.8–16.9)
10001551	Alanine aminotransferase increased	54298	6	2.3 (1.0–5.2)
10043414	Therapeutic response decreased	50145	6	2.5 (1.1–5.6)
10048610	Cardiotoxicity	6896	6	18.3 (8.2–40.9)
10040560	Shock	19115	6	6.6 (3.0–14.7)
10002034	Anemia	167009	6	0.7 (0.3–1.7)
10019717	Hepatitis	21811	6	5.8 (2.6–12.9)
10007554	Cardiac failure	69628	6	1.8 (0.8–4.0)
10016825	Flushing	91611	6	1.4 (0.6–3.1)
10015150	Erythema	176486	6	0.7 (0.3–1.6)
10028411	Myalgia	147005	6	0.9 (0.4–1.9)
10012735	Diarrhea	537371	6	0.2 (0.1–0.5)
10005851	Blood urea increased	15500	6	8.1 (3.6–18.2)
10022611	Interstitial lung disease	40243	6	3.1 (1.4–7.0)
10005364	Blood bilirubin increased	24125	6	5.2 (2.3–11.7)
10033295	Overdose	196181	6	0.6 (0.3–1.4)
10044223	Toxic epidermal necrolysis	12781	5	8.2 (3.4–19.8)
10035528	Platelet count decreased	91823	5	1.1 (0.5–2.7)
10024670	Liver disorder	37415	5	2.8 (1.2–6.8)
–	Malaise	387511	5	0.3 (0.1–0.6)
10027209	Meningitis cryptococcal	1367	5	77.1 (32.0–186.1)
10002199	Anaphylactic shock	21099	5	5.0 (2.1–12.0)
10007559	Cardiac failure congestive	76636	5	1.4 (0.6–3.3)
10003481	Aspartate aminotransferase increased	47010	5	2.2 (0.9–5.4)
10066901	Treatment failure	66328	5	1.6 (0.7–3.8)
10024384	Leukopenia	42467	5	2.5 (1.0–5.9)
–	Gastrointestinal disorder	67315	5	1.6 (0.6–3.7)
10037087	Pruritus	294997	5	0.4 (0.1–0.8)
–	Disease recurrence	39816	5	2.6 (1.1–6.3)
10019670	Hepatic function abnormal	30864	5	3.4 (1.4–8.2)
10005191	Blister	46659	5	2.2 (0.9–5.4)

**Table 2:** Number of reports and reporting odds ratios for liposomal amphotericin B (L-AMB) ^*^ Confidence Interval

Preferred term code	Preferred term	Total (n)	Case (n)	Reporting odds ratio (95% CI^*^)
10013709	Drug ineffective	1128032	311	3.6 (3.2–4.0)
–	Off label use	650743	214	4.1 (3.5–4.7)
10021015	Hypokalemia	39093	147	46.2 (39.0–54.8)
–	Death	730753	75	1.1 (0.9–1.4)
10062237	Renal impairment	70466	61	10.0 (7.7–12.9)
10013968	Dyspnea	486578	59	1.4 (1.1–1.8)
10005483	Blood creatinine increased	57873	58	11.6 (8.9–15.0)
10037660	Pyrexia	299290	54	2.0 (1.6–2.7)
10033661	Pancytopenia	47122	50	12.2 (9.2–16.1)
10069339	Acute kidney injury	125590	43	3.9 (2.9–5.3)
10003481	Aspartate aminotransferase increased	47010	42	10.2 (7.5–13.9)
10038435	Renal failure	122048	38	3.5 (2.6–4.9)
10001551	Alanine aminotransferase increased	54298	38	8.0 (5.8–11.0)
10028813	Nausea	674394	37	0.6 (0.4–0.8)
10035664	Pneumonia	271408	37	1.5 (1.1–2.1)
–	Condition aggravated	240861	36	1.7 (1.2–2.3)
10040047	Sepsis	96944	35	4.1 (2.9–5.7)
10038695	Respiratory failure	63850	35	6.2 (4.4–8.7)
10047700	Vomiting	396955	34	1.0 (0.7–1.3)
10035528	Platelet count decreased	91823	34	4.2 (3.0–5.9)
10005724	Blood potassium decreased	26225	32	13.9 (9.8–19.7)
–	Renal failure acute	43092	32	8.4 (5.9–12.0)
10037844	Rash	358880	31	1.0 (0.7–1.4)
10021097	Hypotension	173419	30	1.9 (1.4–2.8)
10051118	Drug ineffective for unapproved indication	43837	30	7.8 (5.4–11.1)
10051792	Infusion-related reaction	52388	29	6.3 (4.3–9.0)
10002034	Anemia	167009	29	2.0 (1.4–2.8)
10007515	Cardiac arrest	73279	27	4.2 (2.8–6.1)
10005364	Blood bilirubin increased	24125	26	12.2 (8.3–18.0)
10003988	Back pain	202606	25	1.4 (0.9–2.0)
10008479	Chest pain	163743	25	1.7 (1.2–2.5)
10024690	Liver function test abnormal	27025	24	10.0 (6.7–15.0)
10059570	Blood alkaline phosphatase increased	22746	23	11.4 (7.6–17.2)
–	Multi-organ failure	17736	23	14.7 (9.7–22.1)
10040560	Shock	19115	23	13.6 (9.0–20.5)
10005851	Blood urea increased	15500	23	16.8 (11.1–25.3)
–	General physical health deterioration	91284	23	2.8 (1.9–4.3)
10012735	Diarrhea	537371	23	0.5 (0.3–0.7)
10021027	Hypomagnesemia	11470	21	20.7 (13.4–31.8)
10020646	Hyperkalemia	29887	21	7.9 (5.1–12.2)
10007554	Cardiac failure	69628	21	3.4 (2.2–5.2)
10040070	Septic shock	36043	21	6.6 (4.3–10.1)
10013442	Disseminated intravascular coagulation	12728	21	18.6 (12.1–28.7)
10019670	Hepatic function abnormal	30864	21	7.7 (5.0–11.8)
10006473	Bronchopulmonary aspergillosis	6385	21	37.2 (24.2–57.3)
10024670	Liver disorder	37415	20	6.0 (3.9–9.4)
–	Chills	102188	20	2.2 (1.4–3.4)
10038537	Renal tubular disorder	2575	20	88.2 (56.7–137.4)
10018884	Hemoglobin decreased	90933	20	2.5 (1.6–3.8)
10038428	Renal disorder	40451	19	5.3 (3.4–8.3)
10002198	Anaphylactic reaction	44463	19	4.8 (3.1–7.6)
–	Drug interaction	138348	18	1.5 (0.9–2.3)
10029354	Neutropenia	112363	17	1.7 (1.0–2.7)
–	Headache	541085	17	0.3 (0.2–0.6)
10008635	Cholestasis	16115	16	11.2 (6.8–18.3)
–	Disease progression	98477	16	1.8 (1.1–3.0)
10043071	Tachycardia	76652	16	2.3 (1.4–3.8)
10033318	Oxygen saturation decreased	45423	16	4.0 (2.4–6.5)
10033647	Pancreatitis acute	18754	16	9.6 (5.9–15.7)
10038687	Respiratory distress	24185	15	7.0 (4.2–11.6)
10043554	Thrombocytopenia	94721	15	1.8 (1.1–2.9)
10012373	Depressed level of consciousness	34709	15	4.8 (2.9–8.1)
10077361	Multiple organ dysfunction syndrome	20960	15	8.0 (4.8–13.4)
–	Convulsion	57452	15	2.9 (1.8–4.9)
10005734	Blood pressure decreased	57383	14	2.7 (1.6–4.6)
10047942	White blood cell count decreased	93344	14	1.7 (1.0–2.8)
10029155	Nephropathy toxic	8956	14	17.6 (10.4–29.8)
10017533	Fungal infection	28546	13	5.1 (3.0–8.8)
10065553	Bone marrow failure	19108	13	7.6 (4.4–13.2)
10000081	Abdominal pain	198738	13	0.7 (0.4–1.3)
–	Blood magnesium decreased	7302	13	20.0 (11.6–34.6)
10017693	Gamma-glutamyltransferase increased	20209	13	7.2 (4.2–12.5)
10003119	Arrhythmia	42283	12	3.2 (1.8–5.6)
10019075	Hallucination, visual	13307	12	10.1 (5.7–17.9)
10035598	Pleural effusion	53488	12	2.5 (1.4–4.4)
10016825	Flushing	91611	12	1.5 (0.8–2.6)
–	Malaise	387511	12	0.3 (0.2–0.6)
10002199	Anaphylactic shock	21099	12	6.4 (3.6–11.3)
10022611	Interstitial lung disease	40243	12	3.3 (1.9–5.9)
10007617	Cardio-respiratory arrest	38355	12	3.5 (2.0–6.2)
10020642	Hyperhidrosis	113791	12	1.2 (0.7–2.1)
10018838	Hematocrit decreased	18392	12	7.3 (4.1–12.9)
10020772	Hypertension	182177	12	0.7 (0.4–1.3)
10030095	Edema	45676	11	2.7 (1.5–4.9)
10076573	Wrong technique in the product usage process	164412	11	0.7 (0.4–1.3)
10065042	Immune reconstitution inflammatory syndrome	4357	11	28.4 (15.7–51.4)
10015150	Erythema	176486	11	0.7 (0.4–1.3)
10023126	Jaundice	24332	11	5.1 (2.8–9.2)
10003549	Asthenia	323379	11	0.4 (0.2–0.7)
10076476	Product use in unapproved indication	180756	11	0.7 (0.4–1.2)
10038540	Renal tubular necrosis	8395	11	14.7 (8.1–26.6)
10020751	Hypersensitivity	159049	10	0.7 (0.4–1.3)
10038669	Respiratory arrest	25743	10	4.3 (2.3–8.1)
10038535	Renal tubular acidosis	1767	10	63.8 (34.2–118.9)
10020635	Hyperglycemia	31775	10	3.5 (1.9–6.6)
10005470	Blood creatine phosphokinase increased	26696	10	4.2 (2.3–7.8)
10029331	Neuropathy peripheral	77101	10	1.4 (0.8–2.7)
10001052	Acute respiratory distress syndrome	15376	10	7.3 (3.9–13.6)
10000880	Acute myeloid leukemia	13325	10	8.4 (4.5–15.7)
10011224	Cough	234271	10	0.5 (0.3–0.9)
10005725	Blood potassium increased	14240	10	7.9 (4.2–14.7)
10076309	Product use issue	152105	10	0.7 (0.4–1.4)
–	Pain	540553	10	0.2 (0.1–0.4)

**Figure 1:**
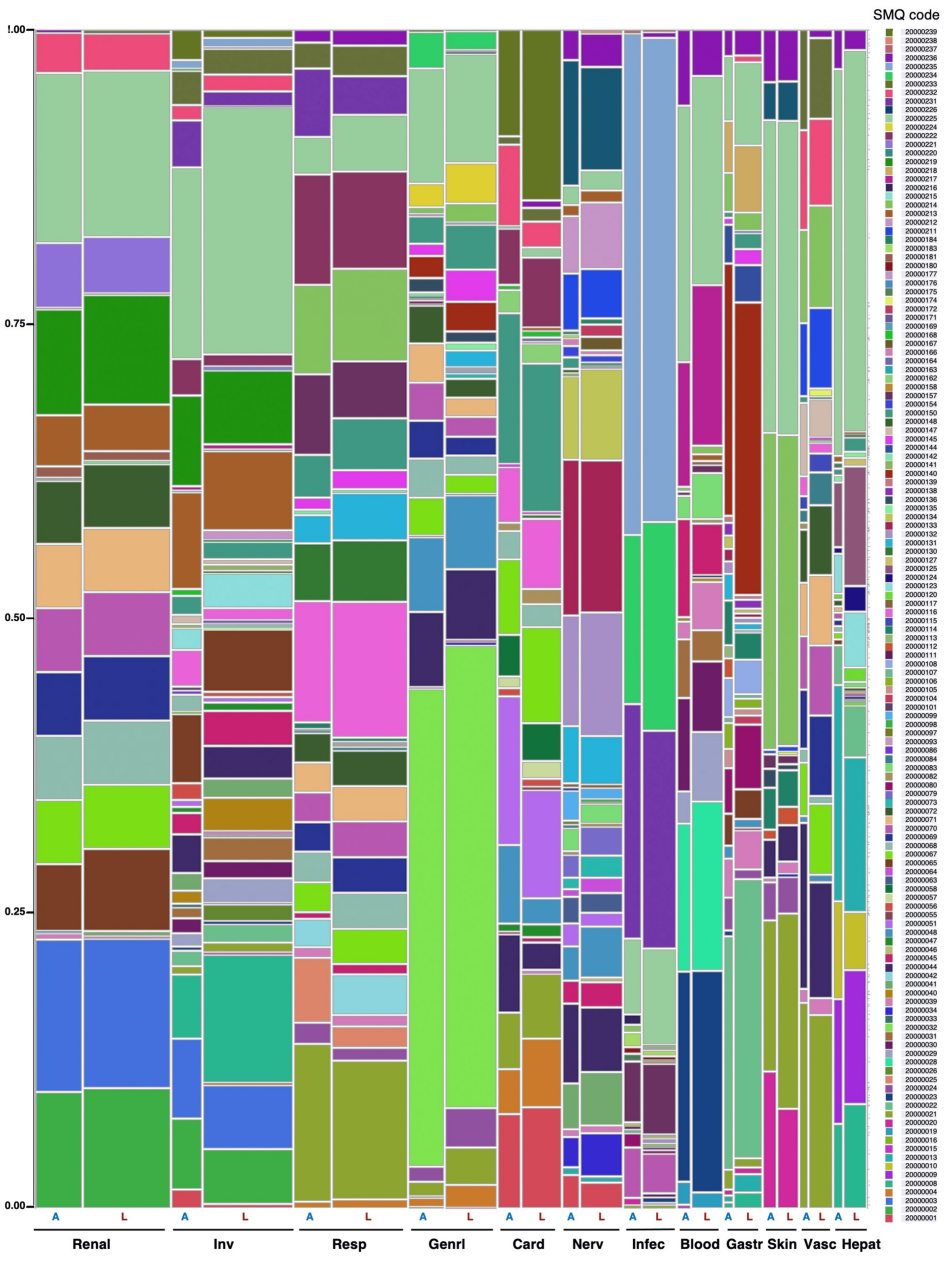
The organ-specific adverse event profiles of AMPH-B and L-AMB based on standardized MedDRA queries (SMQs) The plot is divided into rectangles where each vertical length represents the proportion of each level of the Y variable within each level of the X variable AMPH-B: Amphotericin B deoxycholate; L-AMB: liposomal amphotericin B deoxycholate

Adverse events were classified using SMQs, and the two formulations were compared by organ based on the SOC. The top 12 SOCs with p-values less than 5% are shown in Figure 1 and Table 3.

**Table 3:** List of standardized MedDRA* queries (SMQs) related to AMPH-B and L-AMB ^*^ the Medical Dictionary for Regulatory Activities SIADH: Syndrome of inappropriate antidiuretic hormone secretion; AMPH-B: amphotericin B deoxycholate; L-AMB: liposomal amphotericin B deoxycholate

Code	Standardized MedDRA^*^ queries (SMQs)	Code	Standardized MedDRA^*^ queries (SMQs)
20000001	Torsade de pointes/QT prolongation	20000115	Thrombophlebitis
20000002	Rhabdomyolysis/myopathy	20000116	Acute central respiratory depression
20000003	Acute renal failure	20000117	Psychosis and psychotic disorders
20000004	Cardiac failure	20000120	Infectious biliary disorders
20000008	Liver-related investigations, signs, and symptoms	20000123	Biliary system-related investigations, signs, and symptoms
20000009	Cholestasis and jaundice of hepatic origin	20000124	Gallbladder-related disorders
20000010	Hepatitis, non-infectious	20000125	Biliary tract disorders
20000013	Hepatic failure, fibrosis and cirrhosis and other liver damage-related conditions	20000127	Gallstone-related disorders
20000015	Liver-related coagulation and bleeding disturbances	20000130	Pulmonary hypertension
20000016	Liver infections	20000131	Guillain-Barre syndrome
20000019	Hemolytic disorders	20000132	Noninfectious encephalitis
20000020	Severe cutaneous adverse reactions	20000133	Noninfectious encephalopathy/delirium
20000021	Anaphylactic reaction	20000134	Noninfectious meningitis
20000022	Acute pancreatitis	20000135	Accidents and injuries
20000023	Agranulocytosis	20000136	Extravasation events (injections, infusions and implants)
20000024	Angioedema	20000138	Gastrointestinal nonspecific inflammation
20000025	Asthma/bronchospasm	20000139	Gastrointestinal nonspecific dysfunction
20000026	Dyslipidemia	20000140	Gastrointestinal nonspecific symptoms and therapeutic procedures
20000028	Hematopoietic cytopenias affecting more than one type of blood cell	20000141	Hyponatremia/SIADH
20000029	Hematopoietic erythropenia	20000142	Hostility/aggression
20000030	Hematopoietic leukopenia	20000144	Ischemic colitis
20000031	Hematopoietic thrombocytopenia	20000145	Hemodynamic edema, effusions, and fluid overload
20000032	Lack of efficacy/effect	20000147	Hypertension
20000033	Lactic acidosis	20000148	Optic nerve disorders
20000034	Peripheral neuropathy	20000150	Cardiomyopathy
20000039	Hemorrhage terms (excl laboratory terms)	20000154	Demyelination
20000040	Hemorrhage laboratory terms	20000157	Eosinophilic pneumonia
20000041	Hyperglycemia/new onset diabetes mellitus	20000158	Retinal disorders
20000042	Interstitial lung disease	20000162	Cardiac arrhythmia terms, nonspecific
20000044	Neuroleptic malignant syndrome	20000163	Bradyarrhythmia terms, nonspecific
20000045	Systemic lupus erythematosus	20000164	Tachyarrhythmia terms, nonspecific
20000046	Taste and smell disorders	20000166	Conditions associated with central nervous system hemorrhages and cerebrovascular accidents
20000047	Myocardial infarction	20000167	Depression (excl suicide and self-injury)
20000048	Anticholinergic syndrome	20000168	Other ischemic heart disease
20000051	Arrhythmia-related investigations, signs and symptoms	20000169	Premalignant disorders, general conditions and other site-specific disorders
20000055	Disorders of sinus node function	20000171	Hearing impairment
20000056	Conduction defects	20000172	Vestibular disorders
20000057	Supraventricular tachyarrhythmias	20000174	Vasculitis
20000058	Ventricular tachyarrhythmias	20000175	Conjunctival disorders
20000063	Ischemic central nervous system vascular conditions	20000176	Lacrimal disorders
20000064	Hemorrhagic central nervous system vascular conditions	20000177	Lipodystrophy
20000065	Retroperitoneal fibrosis	20000180	Osteonecrosis
20000067	Shock-associated circulatory or cardiac conditions (excl torsade de pointes)	20000181	Renovascular disorders
20000068	Torsade de pointes, shock-associated conditions	20000183	Ocular infections
20000069	Hypovolemic shock conditions	20000184	Ocular motility disorders
20000070	Toxic-septic shock conditions	20000211	Hypotonic-hyporesponsive episode
20000071	Anaphylactic/anaphylactoid shock conditions	20000212	Generalized convulsive seizures following immunization
20000072	Hypoglycemic and neurogenic shock conditions	20000213	Chronic kidney disease
20000073	Dementia	20000214	Hypersensitivity
20000079	Convulsions	20000215	Malignant lymphomas
20000080	Pseudomembranous colitis	20000216	Arthritis
20000082	Embolic and thrombotic events, arterial	20000217	Myelodysplastic syndrome
20000083	Embolic and thrombotic events, vessel type unspecified and mixed arterial and venous	20000218	Noninfectious diarrhea
20000084	Embolic and thrombotic events, venous	20000219	Tumor lysis syndrome
20000086	Blood premalignant disorders	20000220	Proteinuria
20000093	Malignancy-related therapeutic and diagnostic procedures	20000221	Tubulointerstitial diseases
20000097	Dyskinesia	20000222	Respiratory failure
20000098	Dystonia	20000224	Medication errors
20000099	Parkinson-like events	20000225	Drug reaction with eosinophilia and systemic symptoms syndrome
20000101	Drug abuse and dependence	20000226	Hypoglycemia
20000104	Gastrointestinal perforation, ulcer, hemorrhage, obstruction non-specific findings/procedures	20000231	Infective pneumonia
20000105	Gastrointestinal obstruction	20000232	Dehydration
20000106	Gastrointestinal ulceration	20000233	Hypokalemia
20000107	Gastrointestinal perforation	20000234	Sepsis
20000108	Gastrointestinal hemorrhage	20000235	Opportunistic infections
20000111	Oropharyngeal infections	20000236	Immune-mediated/autoimmune disorders
20000112	Oropharyngeal allergic conditions	20000237	COVID-19
20000113	Gingival disorders	20000238	Sexual dysfunction
20000114	Oropharyngeal conditions (excl neoplasms, infections and allergies)	20000239	Noninfectious myocarditis/pericarditis

The results of the Pearson’s chi-square test indicate that p-values less than 5% were obtained for the following SOCs: “Inv” < 0.0001, “Resp” = 0.0090, “Genrl” < 0.0001, “Card” < 0.0001, and “Blood” = 0.0427. No statistically significant difference was observed in the SMQ profile of adverse events in “Renal (p = 0.9302),” ”Nerv (nervous system disorder) (p = 0.6775),” “Infec (infections and infestations) (p = 0.4707),” and “Hepat (p = 0.5828)” of SOCs between the L-AMB and AMPH-B formulations (Table 4).

**Table 4:** Comparison of SMQ profiles of amphotericin B and liposomal amphotericin B in each SOC classification ^*^ p < 0.05 SMQ: Standardized MedDRA query; SOC: system organ class

System organ class	Abbreviation	Total (n)	Amphotericin B (AMPH-B, n)	Liposomal Amphotericin B (L-AMB, n)	p-value
Renal and urinary disorders	Renal	2412	848	1564	0.9302
Investigations	Inv	2153	548	1605	<0.0001^*^
Respiratory, thoracic and mediastinal disorders	Resp	2029	671	1358	0.0090^ *^
General disorders and administration site conditions	Genrl	1566	646	920	<0.0001^*^
Cardiac disorders	Card	1142	421	721	<0.0001^*^
Nervous system disorders	Nerv	1075	310	765	0.6775
Infections and infestations	Infec	943	327	616	0.4707
Blood and lymphatic system disorders	Blood	827	263	564	0.0427^ *^
Gastrointestinal disorders	Gastr	693	182	511	0.1105
Skin and subcutaneous tissue disorders	Skin	636	249	387	0.9233
Vascular disorders	Vasc	614	177	437	0.8023
Hepatobiliary disorders	Hepat	585	180	405	0.5828
Metabolism and nutrition disorders	Metab	521	160	361	0.0703
Psychiatric disorders	Psych	369	104	265	0.8963
Immune system disorders	Immun	259	91	168	0.9434
Injury, poisoning and procedural complications	Inj&P	241	90	151	0.0875
Musculoskeletal and connective tissue disorders	Musc	177	67	110	0.3746
Eye disorders	Eye	166	35	131	0.9554
Neoplasms benign, malignant and unspecified (incl cysts and polyps)	Neopl	129	45	84	0.2326
Surgical and medical procedures	Surg	54	21	33	0.0548
Ear and labyrinth disorders	Ear	25	5	20	0.0303^ *^
Pregnancy, puerperium and perinatal conditions	Preg	13	8	5	0.7092
Congenital, familial and genetic disorders	Cong	10	2	8	0.5982
Product issues	Prod	5	1	4	0.0821
Reproductive system and breast disorders	Repro	2	2	0	-
Social circumstances	SocCI	1	1	0	-

## Discussion

AMPH-B has severe side effects, such as renal dysfunction, hypokalemia, fever during intravenous administration, chills, nausea, and vomiting [1-5]. Therefore, the patient’s condition should be constantly monitored after drug administration, and the dosage should be adjusted according to the severity of the side effects. L-AMB is a liposomal formulation that was developed with relatively fewer side effects and the same antifungal activity as AMPH-B. L-AMB reduces renal dysfunction by encapsulating AMPH-B within liposomes, which limits leakage from capillaries and transfer to tissue cells while facilitating transfer to infected foci with increased vascular permeability. 

The incidence of hypertension, hypotension, tachycardia, hypoxemia, hypokalemia, and various events associated with impaired renal function is lower with L-AMB than with AMPH-B [4]. Furthermore, acute side effects, such as chills and fever, are reduced by half upon intravenous injection of L-AMB compared to conventional AMPH-B preparation [5]. A meta-analysis revealed that L-AMB reduced nephrotoxicity [7]. L-AMB has been shown to be significantly less toxic than amphotericin B deoxycholate; however, adverse events may still occur [15]. According to the package insert statement, adverse events associated with L-AMB include renal dysfunction, hypokalemia, and hypomagnesemia [3,4]. The fever and hypokalemia reported in previous studies and in the package inserts should be noted, as they were also observed in reports from the FAERS database.

Interestingly, no statistically significant difference was observed in the SMQ profile of adverse events in the renal SOC between the L-AMB and AMPH-B formulations in this study. We consider this result in conjunction with previous studies showing a lower risk of renal impairment with L-AMB than with AMPH-B. L-AMB is approved for use at higher doses than AMPH-B [3,4,16]. Therefore, the absence of significant differences in SMQ profiles when L-AMB was administered at higher doses than AMPH-B suggests that L-AMB is better tolerated by the kidney than AMPH-B. These results may reassure clinicians about the safety of L-AMB in relation to renal function.

According to our study findings, SOCs with statistically significant differences were “Inv,” “Resp,” “Genrl,” “Card,” and “Blood.” In the “Inv” SOC, L-AMB had a higher percentage of reports of liver-related investigations, signs, and symptoms (SMQ: 20000008) and drug reaction with eosinophilia and systemic symptoms syndrome (SMQ: 20000225) than AMPH-B. Further speculations on the reasons for this higher percentage is difficult. Hypokalemia is a well-known adverse event of AMPH-B [1-3]. The SOCs of AMPH-B and L-AMB were compared in the present study, but there was no marked difference in incidence in the rate of hypokalemia (SMQ: 20000233) with the SOCs of either formulation (data not shown).

Despite the broad-spectrum bactericidal activity of AMPH-B, its clinical use has been affected by limitations such as parenteral administration, injection-related reactions, acute and chronic toxicity, and dose limitations. L-AMB can be used for longer periods and at higher doses than AMPH-B, thus representing a major advance in the treatment of invasive fungal infections [1]. This study compares adverse event reports during intravenous administration of L-AMB (Ambisome®, n=1591) and AMPH-B (Fungizone®, n=848). Adverse events of other dosage forms such as amphotericin B lipid complex (ABLC, Abelcet® [17]) and amphotericin B colloidal dispersion (ABCD, Amphocil® [18] and Amphotec® [19]) have been reported in the FAERS. ABCD, which contains uniform disk-shaped particles, was discontinued in 2011 because of its high rate of infusion-related events [1,20]. Adverse events of Abelcet®, Amphocil®, and Amphotec® were reported in six cases, 10 cases, and one case, respectively; the route of administration was entered into the database as intravenous in 6, 7, and 0 of these cases, respectively. Therefore, ABLC and ABCD were excluded from the analysis in this study.

Current lipid-based formulations of amphotericin B such as ABLC and L-AMB show better tolerability and toxicity profiles than AMPH-B but are not without side effects. Lipid-based formulations of amphotericin B have different pharmacological properties compared to AMPH-B [1]. L-AMB and ABLC can be administered at high doses, and their efficacy and toxicity vary by formulation. The guidelines of the Infectious Diseases Society of America state that L-AMB and ABLC have the same spectrum of activity as AMPH-B but different pharmacologic characteristics and frequency of adverse events [21]. ABLC and L-AMB have characteristic pharmacokinetic profiles (Cmax, volume of distribution, and elimination half-life) that determine their efficacy and toxicity, respectively [1], which may influence the final therapeutic outcome. In the future, when enough adverse event reports of ABLC are accumulated in clinical practice, it may be possible to make detailed comparisons with the adverse events of other formulations.

Amphotericin B is the drug of choice for treating many serious fungal infections because it has the widest spectrum of action of all known antifungal agents and the lowest potential for resistance [9]. Recently, an increase in the minimum inhibitory concentration of amphotericin B against *Aspergillus* species was reported. To clarify the global epidemic trends of amphotericin B resistance in clinical *Aspergillus* isolates, the minimum inhibitory concentration of amphotericin B from 2010 to 2020 was systematically evaluated [22]. The results indicated that amphotericin B resistance is more prevalent in *Aspergillus* *terreus* and *Aspergillus flavus* isolates. Some differences were observed in the prevalence of amphotericin B resistance in *Aspergillus* species in various regions. In the present study, we did not analyze the resistance trends by district for the United States, Asia, and Europe. Future analysis should consider the differences in amphotericin B resistance by region.

This study has several limitations. SRSs are subject to over-reporting, under-reporting, missing data, exclusion of healthy individuals, length of the post-launch period of the drug, and presence of confounding factors. Cases reported in the FAERS database do not always contain sufficient information regarding patient background, such as comorbid conditions and concomitant drug administration, to allow for proper evaluation. For example, invasive fungal infection is a life-threatening complication that occurs after allogeneic hematopoietic cell transplantation, with *Candida* and *Aspergillus* being the major causative organisms. Patients with primary agranulocytosis or acquired agranulocytosis (e.g., due to toxicity) who develop invasive fungal infections have different characteristics, history, and outcomes compared to other patient groups. In studies involving adverse spontaneous report databases such as FAERS, it is often difficult to obtain accurate patient backgrounds, and no widely accepted method for statistically adjusting for covariates exists. Therefore, invasive fungal infections can be analyzed only after a sufficient number of cases have been accumulated. Further epidemiologic studies may be needed to confirm the results of the present study; these issues must be fully considered when analyzing drug safety using FAERS data. It would be interesting to investigate the relationship between dose and adverse event occurrence. Although FAERS has a dose entry, it was not considered in this study because many reports contained missing or inaccurate dose calculations. A more detailed analysis focusing on these factors will be the subject of future research.

## Conclusions

Based on real-world data from FAERS, the adverse event profiles of AMPH-B and L-AMB were generated and compared. The SMQ profile of adverse events in renal SOC showed no statistically significant difference between the L-AMB and AMPH-B formulations. Our results suggest that L-AMB is more tolerated by the kidneys than AMPH-B.
